# Impact of Mesh and Fixation on Chronic Inguinal Pain in Lichtenstein Hernia Repair: 5-Year Outcomes from the Finn Mesh Study

**DOI:** 10.1007/s00268-020-05835-1

**Published:** 2020-10-24

**Authors:** M. Matikainen, J. Vironen, J. Kössi, T. Hulmi, M. Hertsi, T. Rantanen, H. Paajanen

**Affiliations:** 1 North-Karelia Central Hospital, Joensuu, Finland; 2 Helsinki University Hospital, Helsinki, Finland; 3 Päijät-Häme Central Hospital, Lahti, Finland; 4 Savonlinna Central Hospital, Savonlinna, Finland; 5 Kuopio University Hospital, Kuopio, Finland; 6 Finland and Eastern University of Finland, Kuopio, Finland

## Abstract

**Objective:**

To find out the mesh fixation technique that minimises chronic pain in Lichtenstein hernioplasty.

**Summary background data:**

Mesh fixation may affect chronic pain and recurrence after inguinal hernia surgery, but long-term results of comparative trials are lacking.

**Methods:**

Lichtenstein hernioplasty was performed under local anaesthesia on 625 patients in day care units. The patients were randomised to receive either a cyanoacrylate glue (*n* = 216), self-gripping mesh (*n* = 202) or non-absorbable 3–0 polypropylene sutures (*n* = 216) for the fixation of mesh. A standardised telephone interview or postal questionnaire was conducted 5 years after the index operation. The patients with complaints suggesting recurrence or chronic pain (visual analogue scale ≥ 3, 0–10) were examined clinically. The rate of occasional pain, chronic severe pain, recurrence, re-operations, daily use of analgesics, overall patient satisfaction and sensation of a foreign object were recorded.

**Results:**

A total of 82% of patients (*n* = 514) completed the 5-year audit including 177, 167 and 170 patients in the glue, self-fixation and suture groups, respectively. There were no significant differences in the incidence of pain (7–8%), operated recurrences (2–4%), overall re-operations (4–5%), need for analgesics (1–2%), patient’s satisfaction (93–97%) or in the feeling of a foreign object (11–18%) between the study groups.

**Conclusion:**

The choice of the mesh or fixation method had no effect on the overall long-term outcome, pain or recurrence of hernia. Less penetrating fixation (glue or self-gripping mesh) is a safe option for the fixation of mesh in Lichtenstein hernia repair.

## Introduction

During the pre-mesh era, a great number of patients suffered from prolonged pain reaction due to tension caused by sutures and they had more recurrences when compared to the Lichtenstein method [1, 2]. The number of recurrences has been reduced to 1–5% in open inguinal hernia surgery owing to the use of synthetic meshes [1]. The main drawback of modern inguinal hernia repair is still chronic pain, which may affect 10–30% of patients after surgery [3]. Chronic inguinal pain is a multifaceted complication which can be due to various surgery and patient dependent factors. The mesh material itself may cause local nerve irritation and scar tissue, although the hernioplasty performed with mesh is tension-free.

In the original Lichtenstein method, the mesh was fixed using non-absorbable 3-0 sutures [1]. Later this method has gone through several modifications all aiming for easier fixation and better outcomes. For example, different suture techniques were introduced and absorbable suture materials or glues were used for fixation [4–9]. Furthermore, hernia mesh technology is under constant development. Different weight and porous size meshes, three dimensionally shaped meshes, totally or partially absorbable meshes have been introduced to the market. Despite the industrial efforts, there is still no common agreement on which would be the ideal mesh for inguinal hernia repair [10].

The chronic pain after inguinal hernia surgery may also be due to the penetrating fixation of the mesh causing possible nerve damage or tension caused by the non-absorbable sutures. Therefore, many studies have tried to demonstrate the benefit of non-penetrating fixation such as self-gripping mesh, fibrin sealant or cyanoacrylate glue [5–7, 9, 11). The short-term results of our randomised trial failed to show any benefit of non-penetrating fixation over traditional non-absorbable sutures [8]. The aim of the present study was to compare long-term results, after 5 years of Lichtenstein hernioplasty, using three fixation techniques.

## Methods

### Trial design

This was a randomised, parallel, prospective, multicentre trial in day care units of seven Finnish hospitals. Recruiting and interventions took place from January 2012 to December 2013. The enrolled patients were consecutive from the waiting list. A more detailed description of the methodology has been published previously [8].

### Participants

**Fig. 1:**
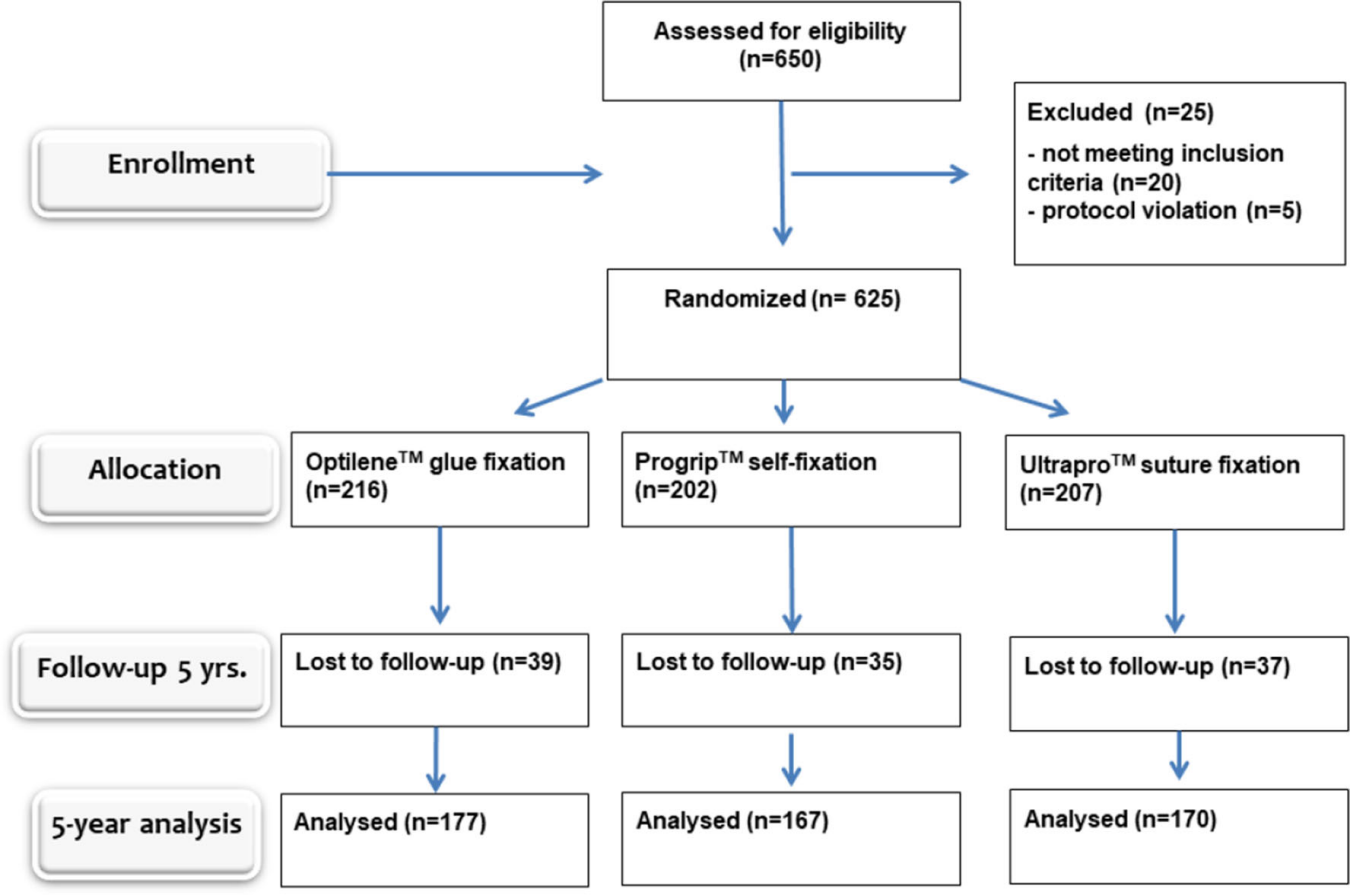
Study flow diagram

The subjects (*n* = 650) were over 18 years old, males and females, with uni- or bilateral primary or recurrent inguinal hernias. The exclusion criteria were femoral hernia, large scrotal hernia, strangulated hernia or patient refusal. The study flow chart is presented in Fig. 1.

### Randomisation

Randomisation was performed separately (block randomisation) in every participating centre as described earlier [8]. Enrolled subjects were assigned to glue fixation, self-gripping or suture fixation according to the allocation designated in the sealed envelope.

### Blinding

This was a single blinded study: The patients were unaware of the fixation method used.

### Interventions

The procedures were performed under local anaesthesia as ambulatory surgery. In cases of bilateral hernias (*n* = 4), each side was treated individually; the second operation began when the first was finished.

The tension-free hernioplasty was performed by using a 9 × 13 cm trimmed lightweight polypropylene mesh (Optilene® mesh 60 g/m^2^, B. Braun, Germany), partly absorbable self-gripping polyester/polylactic mesh (ParietexProGrip®, 14 × 9 cm, 38 g/m^2^, Covidien, USA) or partly absorbable poliglecaprone-25/polypropylene mesh (Ultrapro®, 7.6 × 15 cm, 28 g/m^2^, Ethicon, USA). The fixation of the mesh was done either by a 0.5 ml of butyl-2-cyanoacrylate tissue glue (Histoacryl®, B. Braun, Germany), with a self-fixing mesh (ParietexProgrip®, Covidien, USA) or non-absorbable suture fixation (2-0 Prolene®, Ethicon, USA), as described earlier (8).

### Outcomes

A standardised telephone interview or postal questionnaire was conducted 5 years after the index operation. Computerised patient records in each participating hospital were also re-examined. Groin symptoms were positive, if the patient had experienced pain in the operated inguinal area during the last month. Pain sensation was recorded from 0 to 10 using a visual analogue scale (VAS). Chronic pain was defined as pain rated VAS ≥ 3 limiting normal activity. Patients were also asked whether they had a recurrent hernia or re-operation. All patients complaining of symptoms (pain or bulge) that could be related to a possible recurrence were examined clinically and further with imaging (US or MRI), if necessary. The need for analgesics and for inguinal pain and sensation of a foreign object were also recorded. The primary outcome of this trial was the sensation of pain (VAS ≥ 3) after the 5-year follow-up. Secondary outcomes were recurrences, re-operations for a recurrent hernia or intractable pain, the need for analgesics or a sensation of a foreign object.

### Statistical analysis

Statistical analysis was carried out using Statistical Package for the Social Sciences (SPSS) version 22.0 for Windows (IBM SPSS Statistic 22.0, USA). For categorical variables, we used Pearson’s Chi-squared test or Fisher’s exact test, for numerical variables independent samples *t*-test or Mann–Whitney *U* test.

A *P* < 0.05 was considered statistically significant. Multiple dependent variables (VAS) measured at multiple time periods (at 1 day, 7 days, 30 days, 1 year and 5 years after the operation) were modelled using the GLM repeated measures procedure. An assessment of the normality of data was performed using SPSS statistic (explore command).

## Results

All randomised patients received the intended treatment with 216, 202 and 207 patients in each group (Fig. 1). A total of 82% of patients completed the 5-year follow-up: 177 patients in the glue group, 167 patients in the self-fixation mesh group and 170 patients in the suture group were re-analysed. There were no statistically significant differences in patient characteristics between the study groups at 5 years, which indicates that initial randomisation was successful (Table 1).

**Table 1:** Five-year demographic data presented as absolute numbers (%) or means (±*SD*)

	Glue fixation	Self-gripping mesh	Suture fixation	*P*-value
	(*n* = 177)	(*n* = 167)	(*n* = 170)	
Male/female	160/17 (90/10)	158/9 (95/5)	161/9 (95/5)	0.1167
Mean age	64 (14)	61 (14)	62 (13)	0.0826
Left/right	67/110 (38/62)	75/92 (45/55)	63/107 (37/63)	0.2685
Direct/indirect	63/101 (36/57)	57/100 (34/60)	50/106 (29/62)	0.4827
Combined	13 (7.3)	10 (6.0)	14 (8.2)	0.7242
Recurrent	4 (2.2)	11 (6.6)	9 (5.3)	0.1468
Size of defect at operation (cm):				
<1.5	94 (53)	89 (53)	89 (52)	0.9833
1.5–3	51 (29)	46 (28)	51 (30)	0.8835
>3	32 (18)	32 (19)	30 (18)	0.9337

Altogether 16 patients (2.9%) had a recurrent hernia operated during 5-year follow-up with no difference between the study groups (Table 2). All recurrences (11 direct and 5 indirect hernias) were re-operated using total extraperitoneal technique (TEP). A mild scar bulge, needing no surgical intervention, was reported in 24 patients (4.7%) with no difference between the study groups. Other re-operations (except recurrence) were performed in four patients in the glue group (three severe inguinodynia and one removal of lipoma). In the self-gripping mesh group, one patient was re-operated to evacuate postoperative hematoma. In the suture group, two patients were re-operated (one pain and one seroma evacuation). When re-operating due to overwhelming pain, mesh was removed in two patients and an irritated nerve was released or cut in two patients. Inguinal ultrasound was performed in 37 patients during 5-year follow-up (Optilene group *n* = 13, Progripp *n* = 13 and Ultrapro *n* = 11). Ultrasound examination was usually necessary if no obvious recurrence was found during clinical examination (i.e. inguinal pain, obese patient). In cases of severe inguinal pain without a clinical recurrence, magnetic resonance imaging was performed in six patients with negative results.

**Table 2:** Follow-up data (mean ± SD) after 1 and 5 years

	Glue fixation	Self-gripping mesh	Suture fixation	*P*-value
	(*n* = 216)	(*n* = 202)	(*n* = 207)	
1 year:	*n* = 208	*n* = 193	*n* = 198	
Recurrence operated	2 (0.9)	0 (0)	2 (0.9)	0.3833
Bulge	2 (0.9)	2(1.0)	1	0.8207
VAS ≥ 3	20 (9.6)	20 (10)	12 (6.0)	0.2681
Mean pain (VAS)^1^	0.6 ± 1.5	0.6 ± 1.4	0.4 ± 1.3	0.2614
Feeling of foreign object	35 (17)	35 (18)	26 (13)	0.3735
Need of analgesics	9 (4.3)	5 (2.6)	11 (5.6)	0.3384
Not satisfied	14 (6.7)	10 (5.2)	9 (4.5)	0.5974
5 years:	*n* = 177	*n* = 167	*n* = 170	
Recurrence operated	4 (2.2)	7 (4.2)	5 (2.9)	0.5804
Bulge	2 (1.1)	5 (3.0)	4 (2.3)	0.4770
Mean pain (VAS)^1^	0.1 ± 1.3	0.1 ± 1.3	0.1 ± 1.3	0.9999
Feeling of occasional pain	12 (6.8)	18 (11)	16 (9.4)	0.4163
VAS ≥ 3	12 (6.8)	14 (8.4)	14 (8.2)	0.8267
Need of analgesics	3 (1.7)	2 (1.2)	1 (0.6)	0.6305
Feeling of foreign object	27 (15)	30 (18)	18 (11)	0.1516
Not satisfied	14 (6.7)	10 (5.2)	9 (4.5)	0.5874

Percentages are in parentheses

^1^VAS means visual analogue scale

**Fig. 2:**
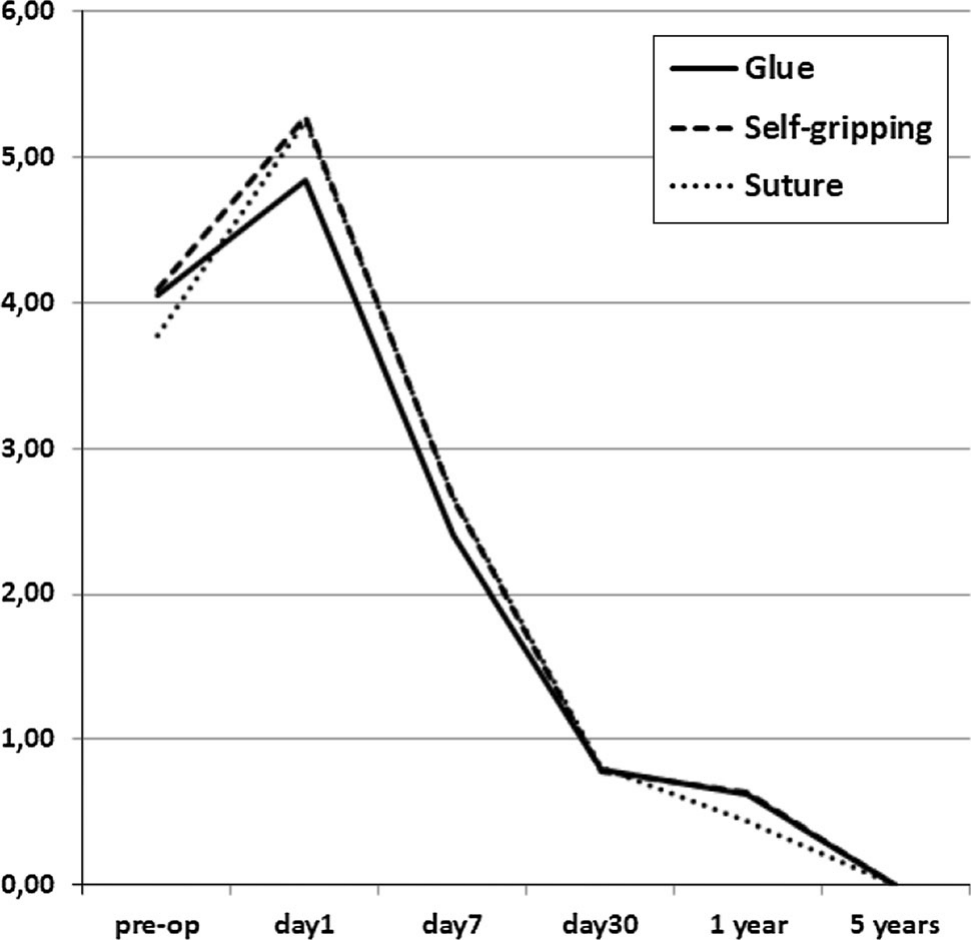
Median pain scores (VAS 0–10) before and after Lichtenstein operation. No statistically significant difference between any of the groups

**Fig. 3:**
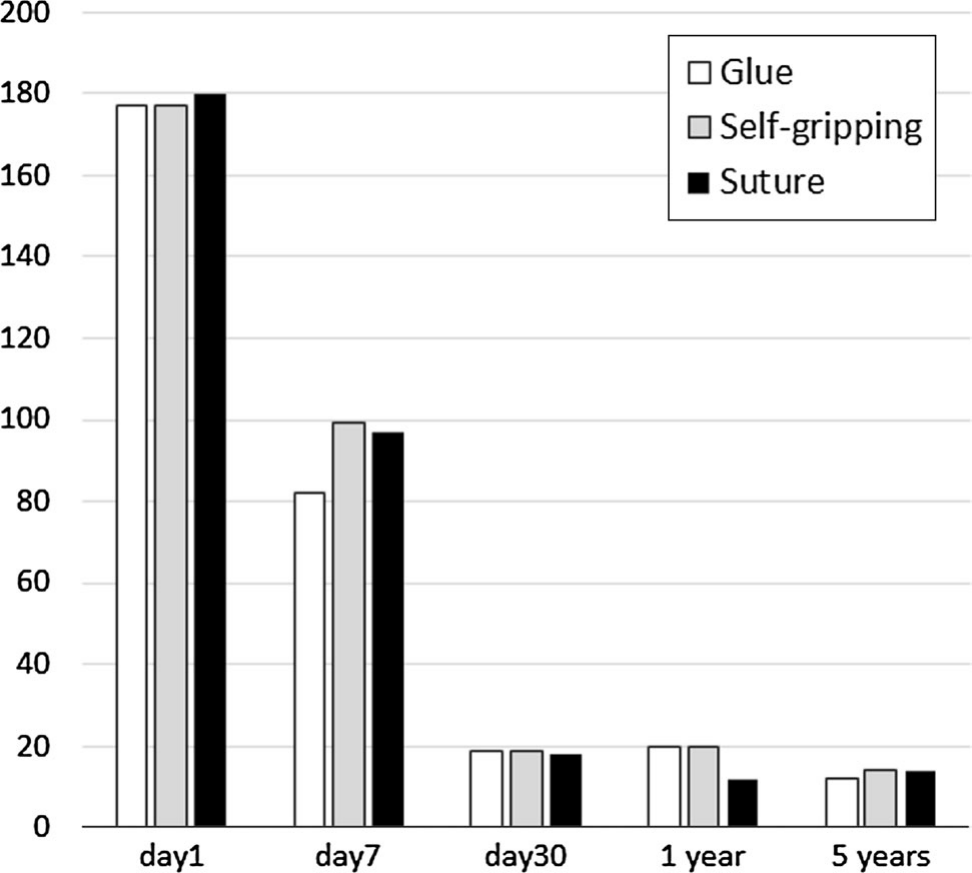
Number of patients having pain scores VAS ≥ 3 after hernia operation. No statistically significant difference between any of the groups

A feeling of a foreign object, in the operated area, was reported in 11–18% of patients. This caused no harm to patients. The number of patients having chronic pain scores ≥ 3 varied between 7–8% (ns), but only five patients needed analgesics temporarily. Figure 2 shows median numeric pain scores, which did not differ between the three study groups. The number of patients having VAS scores ≥ 3 is presented in Fig. 3. The choice of the mesh or fixation method had no effect on the long-term results since there were no differences in any of the studied outcomes between the groups (Table 2, Figs. 2 and 3).

## Discussion

Our hypothesis was that the non-penetrating fixation of mesh would cause less chronic pain than fixation with sutures during 5 years of follow-up, but we found no statistically significant difference in any of the measured parameters. Operation times were shorter when using glue or self-gripping mesh compared to a conventional suture fixation [8]. Also, our previous studies have demonstrated that absorbable or non-absorbable sutures or glue fixation has no effect on chronic pain [4, 12]. The number of re-operations, hernia recurrences or chronic pain was all low in the present study. Meticulous surgical techniques may be more important to decrease in long term the patient’s discomfort than the type of mesh or fixation method.

In line with our results, a previous well-made randomised study by Kim-Fucs et al. [7] comparing cyanoacrylate glue and traditional non-absorbable sutures for mesh fixation reported no significant differences in chronic pain or recurrences at 5-year follow-up. However, several other randomised controlled trials with a follow-up until 12 months have reported decreased chronic pain when using tissue glue compared to traditional suture fixation in Lichtenstein hernioplasty (5, 6, 9). A recent Cochrane systematic review found out that glue fixation of the mesh may decrease chronic pain when compared with suture fixation, but the level of evidence was deemed low and the risk of bias high [11].

In this trial, a total of 19 patients were re-operated on. Fifteen patients had a re-operation due to a recurrent hernia, and four patients had a re-operation due to difficult chronic pain. An open anterior approach was used for patients re-operated on due to pain; two neurectomies and two mesh removals were done. All the re-operated patients had an uneventful recovery from the second surgery. Mesh removal has been proved to be an effective treatment for chronic inguinodynia after Lichtenstein repair [13]. The low number of re-operations may be explained by the fact that all the operations were performed by senior surgeons.

The strength of our study was a long-term follow-up and a prospective randomised design. Furthermore, we reached 82% of patients to follow-up the groin symptoms. The most obvious weakness of our study was that all the patients were not examined clinically, thus affecting the number of total recurrences detected (i.e. minor non-clinical recurrences were not recorded). Also, the use of different meshes may have influenced the results although our previous studies have not demonstrated any difference between different meshes in terms of pain or recurrences [10].

Less fixation does not mean less pain in the long-term follow-up. There was no statistically significant difference in pain scores, recurrence rate of hernias or in the satisfaction for the operation after 5 years between glue fixation, self-gripping mesh or suture fixation. This may give both the surgeon and the patient the option to choose a variety of operating methods to perform open inguinal hernioplasty.
